# Thioredoxin system-mediated regulation of mutant Kras associated pancreatic neoplasia and cancer

**DOI:** 10.18632/oncotarget.21539

**Published:** 2017-10-04

**Authors:** Michelle A. Schultz, Andrew M. Diaz, Sharon Smite, Anna R. Lay, Brian DeCant, Ronald McKinney, Windel E. Mascarinas, Yinglin Xia, Carola Neumann, David Bentrem, David W. Dawson, Paul J. Grippo

**Affiliations:** ^1^ Division of Gastroenterology, Department of Medicine, University of Illinois-Chicago, Chicago IL 60612, USA; ^2^ Department of Surgery, Northwestern University Feinberg School of Medicine, Chicago IL 60611, USA; ^3^ Department of Pathology and Laboratory Medicine and Jonsson Comprehensive Cancer Center, David Geffen School of Medicine at University of California Los Angeles, Los Angeles, CA 90095, USA; ^4^ Department of Pharmacology and Chemical Biology, University of Pittsburgh Medical Center, Pittsburgh PA 15232, USA

**Keywords:** peroxiredoxin-1 (Prdx1), thioredoxin (Txn) system, Kras, pancreatic cancer

## Abstract

Peroxiredoxin-1 (Prdx1), a member of the thioredoxin (Txn) system, is overexpressed and correlates with poor prognosis in pancreatic cancer patients and can suppress Kras signaling through redox-mediated inhibition of ERK and AKT in lung and breast cancer. Its redox function is maintained by Txn and sulfiredoxin (Srxn), and its tumor promoting functions are activated by post-translational modification. We studied the role of the Txn system in pancreatic neoplasia and cancer by determining how it regulates the phosphorylation of Kras effectors and by determining its association with patient survival. We found that elevated Prdx1 nuclear localization significantly correlated with better patient survival. Our data also demonstrate that the expression of the Txn system is dysregulated, with elevated Prdx1 expression and significantly decreased Txn and Srxn expression in pancreatic lesions of targeted mutant Kras mouse models. This correlated with distinct differences in the interconversion of Prdx1 oligomers that affect its ability to regulate ERK and AKT phosphorylation. Our data also suggest that Prdx1 post-translational modification and oligomerization suppress Prdx1 mediated redox regulation of ERK phosphorylation. We observed distinct differences in Txn expression and in the ability of pTyr-Prdx1 to bind to pERK in a PanIN model of pancreatic neoplasia as compared to an IPMN model, indicating a distinct difference in the function of post-translationally modified Prdx1 in cells with less Txn expression. Modified Txn system function and post-translational regulation may therefore play a significant role in pancreatic tumorigenesis by altering Kras effector phosphorylation and inhibiting the tumor suppressive redox functions of Prdx1.

## INTRODUCTION

Improvement of pancreatic cancer therapy requires sensitive early detection techniques and a better understanding of the biology of pancreatic neoplasia. Pancreatic cancer is a devastating disease that is often associated with Kras mutation, which can activate Nrf2 signaling [1]. Peroxiredoxin-1 (Prdx1) is elevated in pancreatic cancer patient tissue and serum and correlates with worse survival [2, 3]. Prdx1 is a member of the Thioredoxin-associated system of antioxidant proteins which is regulated by Nrf2 and Nrf1 [4, 5]. Additional members of the Thioredoxin system include Thioredoxin (Txn), Sulfiredoxin (Srxn), and Thioredoxin Reductase (TxnR) among others [6–9]. Dysregulation of Nrf2 and the Txn system (through post-translational modification) can have significant effects on tumorigenesis and the response to therapy by modifying the ability of these antioxidant proteins to affect transcription, nuclear chaperoning, and the response of cancer cells to oxidative stress associated with chemotherapy and radiation [10–12]. Prdx1 depends on Txn and Srxn to prevent or reverse its overoxidation and peroxidase inactivation [13, 14]. Due to mutant Kras-associated upregulation of Nrf2, Txn system expression may be modified during pancreatic neoplasia [1]. Overoxidation, phosphorylation, and oligomerization of Prdx1 significantly modifies its cofactor and peroxidase functions [15, 16], which will significantly alter its ability to regulate the activity of redox sensitive transcription factors and signaling proteins. In its reduced state Prdx1 can suppress the activity of ERK, AKT, and NF-kB [15–18]. Global knockout of Prdx1 *in vivo* also causes the formation of multiple malignancies in mice [19]. Inhibition of Prdx1 peroxidase activity will therefore alter its tumor suppressive functions. In this study, we characterized changes in the expression and function of the Txn system during pancreatic neoplasia and cancer and investigated its role in regulating mutant Kras associated pancreatic tumorigenesis.

Oxidized and oligomerized Prdx1 can be secreted and may play a role in perpetuating inflammatory signaling [20–22]. Oligomeric Prdx1 exists in several combinations of Prdx1 dimers, including decamers [14, 23–26]. The interconversion of Prdx1 oligomers and the suppression of Prdx1 redox function can be regulated by Tyr phosphorylation of Prdx1. These oxidized and/or phosphorylated oligomers have various functions including nuclear chaperoning and transcriptional regulation. Prdx1 monomers also exist in a reduced or hyperoxized state. Modified Prdx1 post-translational regulation also alters pERK and pAKT signaling in mutant Kras cells. Oxidation of the redox sensitive active site of PTEN (which results in its inactivation via disulfide bond formation), is reversed by Prdx1, resulting in suppression of AKT phosphorylation [17]. ERK signaling is also suppressed by Prdx1 in Kras mutant lung tumorigenesis [18]. Disruption of Prdx1's redox function, either through overoxidation, phosphorylation, or oligomerization could significantly affect the redox-associated regulation of these Kras effectors. We therefore characterized the expression of the Txn system in mutant Kras pancreatic lesions and investigated its role in regulating ERK and AKT phosphorylation. We also investigated the role of these Prdx1 post-translational modifications in altering Prdx1 signaling in mutant Kras neoplastic pancreatic cells. We found that Txn system expression is clearly modified in pancreatic lesions of patients and mice and observed a significant correlation between high Prdx1 nuclear localization and improved patient survival. We also demonstrate that distinct differences in the ability of Prdx1 to regulate ERK and AKT phosphorylation are associated with changes in Prdx1 post-translational modification, oligomerization, and interaction with ERK. Our study therefore demonstrates that dysregulation of Prdx1 and Txn system expression and function enhance pancreatic tumorigenesis by altering Prdx1's ability to suppress ERK and AKT phosphorylation.

## RESULTS

### Upregulation of Prdx1 in human pancreatic cancer patients

**Figure 1:**
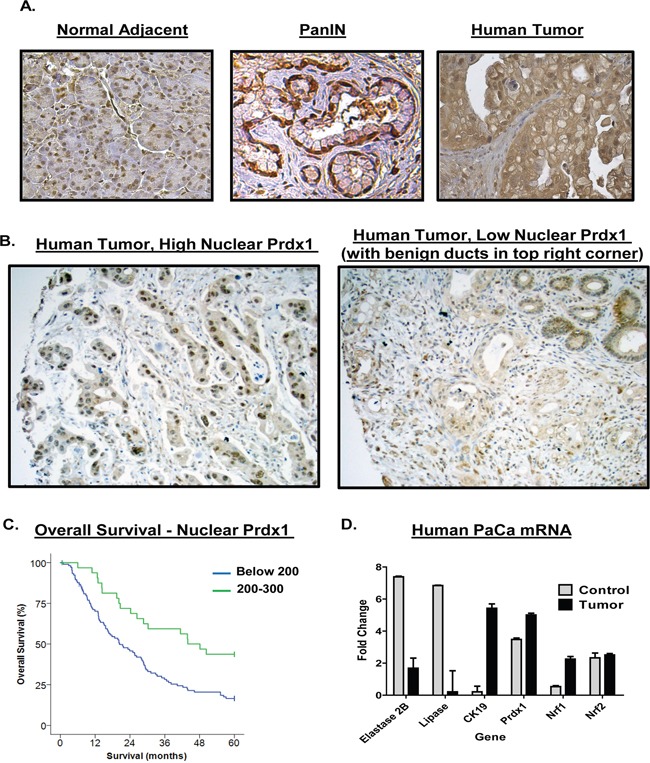
Upregulation of Prdx1 expression in human pancreatic cancer patients **(A)** Human pancreatic cancer patient samples were stained for Prdx1 by immunohistochemistry in normal pancreatic tissue, PanIN, and tumor tissue. (n=60). **(B)** Nuclear Prdx1 levels in human pancreatic cancer patient samples on additional pancreatic cancer tumor array (n=139). **(C)** Overall survival of patients with high and low nuclear Prdx1 expression in tumor array shown in (B). **(D)** mRNA expression of Prdx1, the redox regulating transcription factors Nrf1 and Nrf2, and acinar and ductal markers in tumor tissue was compared using human pancreatic cancer data from Oncomine (normal- n=5, tumor n=10). (See Supplementary Figures 1-3.)

We evaluated the expression of Prdx1 in human pancreatic cancer tissue. We found that overall Prdx1 expression was elevated in tumor tissue as compared to adjacent normal tissue in a pancreatic cancer patient tumor array (n=60) (Figure 1A). Prdx1 staining in dysplastic ducts showed strong nuclear and cytoplasmic Prdx1 staining (Supplementary Figure 3). Additionally, to further investigate the importance of Prdx1 localization in pancreatic cancer patient tumor samples, we examined the correlation between Prdx1 nuclear localization, overall survival, and several clinicopathological parameters in a separate patient tumor array (tumor samples only) (n=139) (Figure 1B). We found that there was a highly significant positive correlation between high nuclear Prdx1 expression and longer survival of patients (p=0.001) (Figure 1C). The median survival of patients with low nuclear Prdx1 expression was 20.4 months and the median survival of patients with high levels of nuclear Prdx1 was 43.92 months (See Supplementary Figures 1 and 2 for additional information). Interestingly, there was also a near significant (p=0.063) association between low nuclear Prdx1 expression and positive lymph node involvement. There was no significant correlation between nuclear Prdx1 expression and age, gender, histologic grade, T-stage, AJCC stage, surgical margins, or tumor size. This data suggests that Prdx1 expression and localization is associated with changes in patient survival in pancreatic cancer.

To determine if mRNA expression of Prdx1 and its transcriptional regulators (Nrf1, Nrf2) is elevated in patients, we searched the Oncomine mRNA database of normal and tumor pancreatic tissue (Figure 1D). We used the Logsdon mRNA data set comparing mRNA levels in microdissected normal pancreatic tissue (n=5) and pancreatic tumors (n=10) [27]. We found that, as compared to normal pancreatic tissue, patient tumors had higher levels of both Prdx1 and Nrf1 mRNA (P= 2.92E-08 and 2.68E-07, respectively (Oncomine P values)). This correlated with changes in the expression of classical acinar and ductal markers (Elastase, Lipase, and CK-19). Interestingly, however, we did not see significant changes in the expression of Nrf2 mRNA between patient normal tissue and tumors. This suggests that on a transcriptional level, Nrf1 expression may also be induced in pancreatic cancer and available to induce Prdx1 expression in pancreatic cancer.

### Expression of the Txn system is dysregulated during pancreatic neoplasia *in vivo*

**Figure 2:**
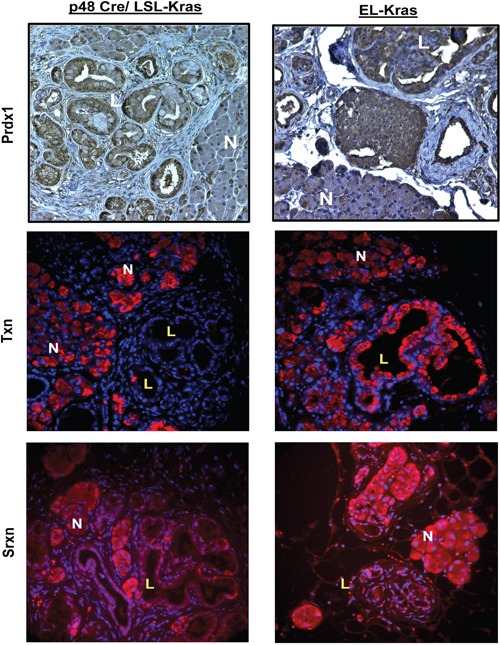
Expression of the Txn system is dysregulated during pancreatic neoplasia *in vivo* KC and EL-Kras mice were stained for Prdx1 (IHC), Txn (IF), and Srxn (IF). Both normal pancreatic tissue and lesions are represented. N=normal tissue and L=lesions in enlarged inset. (n=5/genotype). (Enlarged pictures in Supplementary Figures 4-6.)

To determine if changes in Prdx1, Txn, and Srxn expression correlate with changes observed in early stage disease, we employed mouse models of pancreatic neoplasia resembling PanIN (Pancreatic intraepithelial neoplasia) and IMPN (Intraductal papillary mucinous neoplasm). We evaluated Prdx1, Txn, and Srxn expression in p48-Cre/LSL-Kras (KC; PanIN model) and EL-Kras (IPMN model) mice tissue (Figure 2) (See Supplementary Figures 4-6 for enlarged pictures. See Supplementary Figure 7 for CK19/Txn staining.). There was mild cytoplasmic and nuclear Prdx1 staining in normal acinar cells, while accompanying cells undergoing ADM in these compartments showed increased Prdx1 expression. In both models, total Prdx1 expression was higher in lesions, but there were differences in Prdx1 localization in the normal tissue and lesions of each model. In EL-Kras mice, nuclear Prdx1 levels were similar in normal pancreatic tissue and cystic papillary neoplasms (CPN), while cytoplasmic Prdx1 expression was slightly elevated in the cytoplasm of CPNs. Nuclear Prdx1 levels were lower than cytoplasmic Prdx1 levels in normal tissue and lesions. In KC mice, both nuclear and cytoplasmic Prdx1 expression was higher in PanINs than in normal tissue. In contrast to EL-Kras mice, Prdx1 nuclear levels were higher than cytoplasmic Prdx1 levels in both normal tissue and lesions of KC mice. Lesions of KC mice also had mildly staining mucin. Cells lining reactive ducts in both models also had strong nuclear and cytoplasmic Prdx1 staining. These changes in Prdx1 expression and localization demonstrate that the expression, localization, and function of Prdx1 are modified during pancreatic neoplasia.

We also evaluated Txn and Srxn expression to determine if changes in Prdx1 expression and localization are associated with changes in its ability to be reduced. Interestingly, in KC lesions, Txn expression is undetectable and Srxn expression is primarily located in the nucleus. A similar pattern of Srxn expression was found in EL-Kras CPNs, but Txn expression in lesions was comparable to that of ADM cells in these mice. Our data suggest that Prdx1 may be more oxidized in pancreatic lesions due to decreased expression and modified localization of Txn and Srxn.

### pERK and Prdx1 interactions and Txn system regulation of pERK and pAKT in pancreatic cells

**Figure 3:**
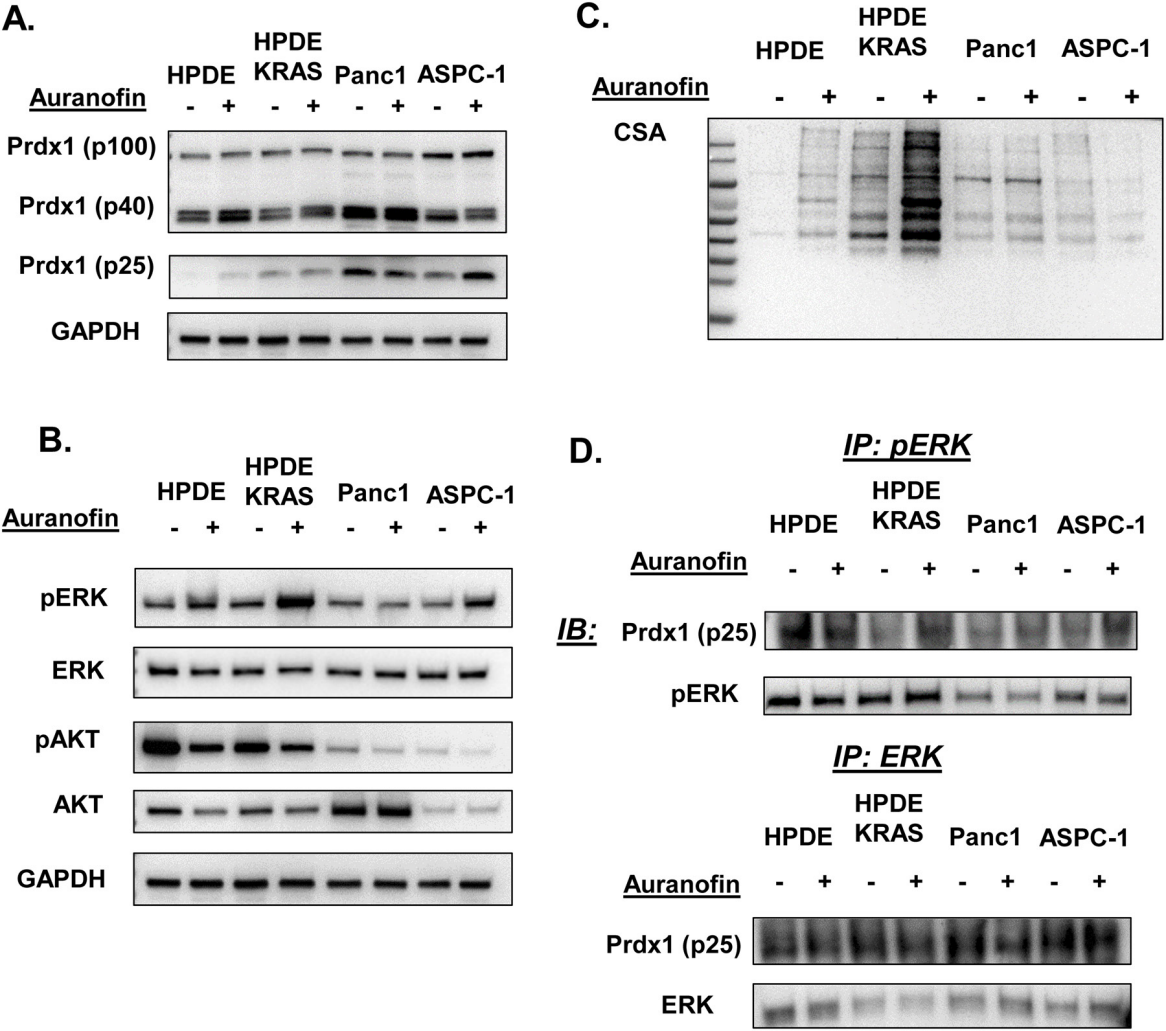
pERK and Prdx1 interactions and Txn system regulation of pERK and pAKT in pancreatic cells The effect of auranofin on Kras effectors and the Txn system was evaluated *in vitro* in normal and mutant Kras ductal cells (HPDE and HPDE-Kras) and in primary tumor derived (Panc1) and metastatic pancreatic cancer cell lines (AsPC-1). Auranofin induced **(A)** Prdx1 expression in non-reducing westerns, **(B)** ERK and AKT phosphorylation, and **(C)** total protein cysteine oxidation (CSA) in non-reducing westerns. **(D)** pERK and total ERK protein was immunoprecipitated then probed for Prdx1 binding in reducing westerns (westerns n=7, immunoprecipitations n=3). (Statistical analysis shown in Supplementary Figures 8 and 9.)

**Figure 4:**
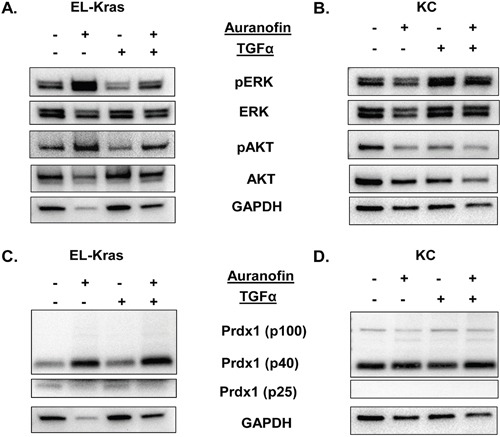
Regulation of the Txn system and its effect on ERK and AKT phosphorylation in primary culture of mutant Kras pancreatic cells Primary pancreatic cells were derived from EL-Kras and KC mice and treated with the TxnR inhibitor auranofin. **(A,B)** Regulation of ERK and AKT phosphorylation by auranofin in (A) EL-Kras and (B) KC mice. **(C,D)** Auranofin induced changes in Prdx1 expression in (C) EL-Kras and (D) KC mice in non-reducing westerns (EL-Kras n=4, KC n=4). (Statistical analysis shown in Supplementary Figures 10 and 11. Supporting Txn and Srxn data shown in Supplementary Figures 11 and 12.)

To further investigate the effect of a dysregulated Txn system in pancreatic neoplasia in the context of mutant Kras, we used the TxnR inhibitor auranofin [28] in human pancreatic ductal and cancer cell lines *in vitro* to determine how oxidation of the Txn system affects ERK and AKT phosphorylation (Figure 3). There was a small but significant (P=0.0169) increase in ERK phosphorylation in response to auranofin in HPDE-Kras cells and similar to KC cells in Figure 4, a significant decrease in AKT phosphorylation in HPDE and Panc1 cells (P=0.0493 and 0.0211 respectively) (See Supplementary Figure 8A). In addition, p25 Prdx1 was differentially affected by auranofin than the p40 Prdx1 homodimer or the p100 Prdx1 oligomer. In metastatic AsPC-1 cells, we also observed a potential Prdx1 decamer (∼250+ kDa) (Supplementary Figure 8C). There was also an increase in p100 Prdx1 levels in AsPC1 cells in response to auranofin. This indicates that changes in Prdx1 oligomerization could be associated with the aggressiveness or progression of the disease. This is further supported by higher basal p100 Prdx1 levels (Figure 3A), high MW p28 Srxn (Supplementary Figure 12) levels, and differential CSA responses (Figure 3C) in Panc1 and ASPC-1 cancer cells as compared to normal ductal HPDE cells and Kras mutant HPDE-Kras cells. Therefore, Prdx1 oligomerization may be differentially regulated in normal and cancer cells and associated with changes in Kras effector phosphorylation.

To determine if Prdx1 can affect ERK and AKT signaling through direct interaction, we immunoprecipitated p/ERK and p/AKT then probed for Prdx1 (Figure 3D). No interactions between Prdx1 and pAKT or total AKT were observed (data not shown). Both pERK and total ERK were bound to p25 Prdx1 in all cell types, although there were no significant changes in binding in response to auranofin in any cell type in total lysate. Additionally, because the p40 Prdx1 homodimer in non-reducing westerns migrates at p25 Prdx1 in reducing westerns, it is possible that both p40 and p25 Prdx1 are bound to ERK in these cells (Supplementary Figure 8B). This suggests that Prdx1 does not affect AKT signaling through direct interaction with pAKT and that p/ERK signaling may be regulated through direct interaction with Prdx1.

### Regulation of the Txn system and its effect on ERK and AKT phosphorylation in primary culture of Kras mutant pancreatic cells

Similarly, we used auranofin and TGFα in primary pancreatic acinar cells to determine how Txn system oxidation affects ERK and AKT phosphorylation (Figure 4) with and without external stimuli. In primary cells from 5 month old EL-Kras and KC mice, we found that the ability of Prdx1 to respond to auranofin correlated with the response of pERK and pAKT to auranofin.

In cells from EL-Kras mice, auranofin enhanced ERK and AKT phosphorylation regardless of the presence of TGFα (Figure 4A and Supplementary Figure 10). In cells from KC mice, pERK did not respond to auranofin, although TGF-α significantly increased ERK phosphorylation (p=0.0121) (Figure 4B). Interestingly, auranofin did suppress the ability of TGF-α to induce ERK phosphorylation. By contrast, TGF-α had no significant effect on AKT phosphorylation in KC cells and pAKT levels were significantly decreased by auranofin (p=0.0193). These data suggest that ERK and AKT phosphorylation are differentially affected by oxidation of the Txn system in IPMN-like EL-Kras and PanIN-like KC pancreatic lesions.

Interestingly, differences in the ability of auranofin to affect ERK and AKT phosphorylation correlated with differences in auranofin's ability to affect levels of oligomerized Prdx1. The p40 Prdx1 homodimer was significantly enhanced in response to auranofin in EL-Kras mice (p≤0.05) indicating increased Prdx1 oxidation, but not in KC mice. This could be associated with auranofin induced changes in the expression or interconversion of Prdx1 oligomers. Moreover, no p100 Prdx1 band was present in cells from EL-Kras mice (Figure 4C), but this oligomerized and oxidized band was present in cells from KC mice (Figure 4D). Txn expression was not affected by auranofin and Srxn signaling is similarly stimulated by auranofin in both models (Supplementary Figures 11 and 12). These differential patterns of Prdx1 oligomer expression and responses to auranofin suggest that changes in Prdx1 oligomerization can have significant effects on oxidative stress associated changes in ERK and AKT phosphorylation in IPMN and PanIN lesions.

### Prdx1 phosphorylation and pERK interactions in EL-Kras and KC mouse tissue

**Figure 5:**
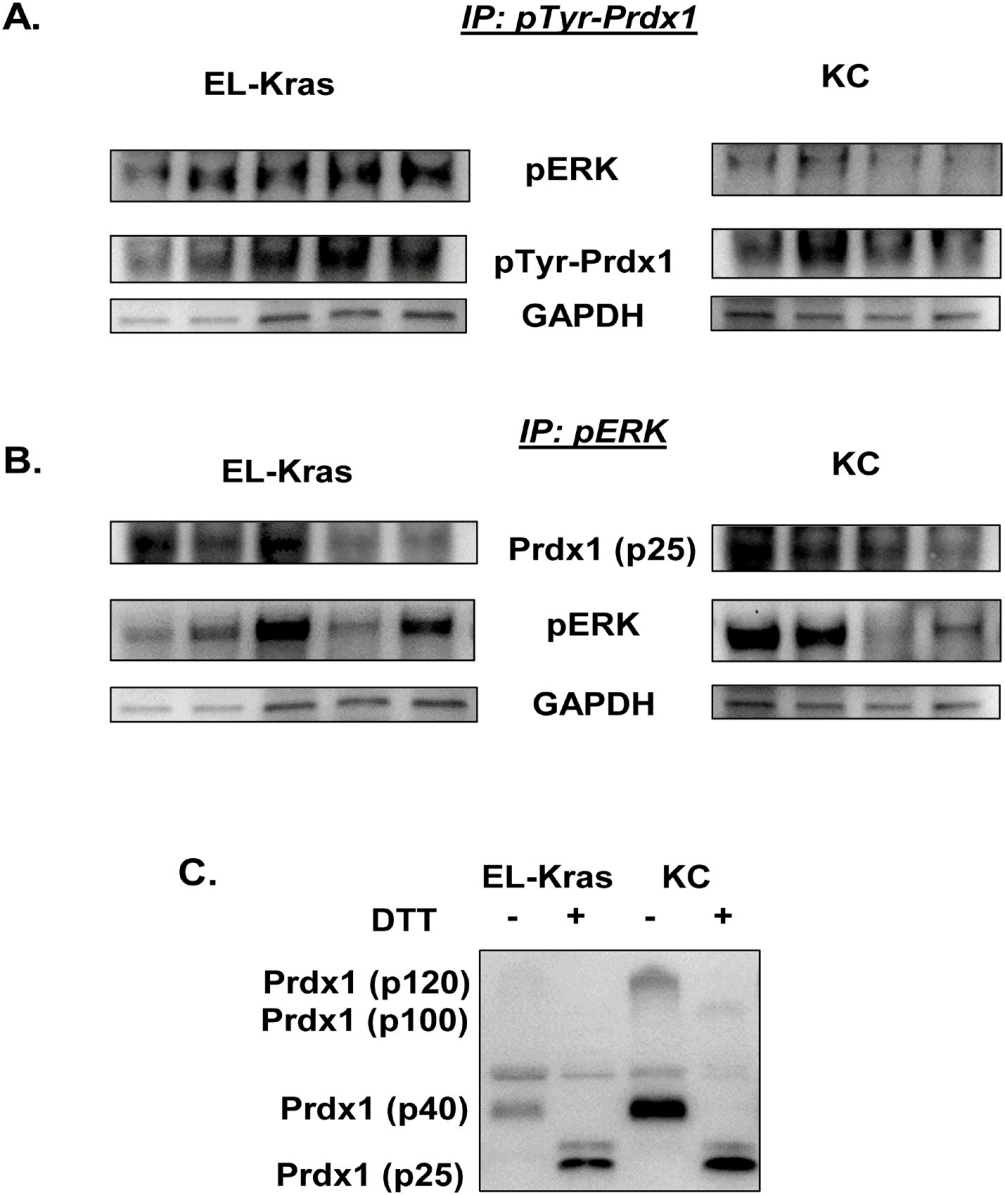
Differential pTyr-Prdx1/ERK interactions in EL-Kras and KC mouse tissue **(A)** Levels of pERK bound to pTyr-Prdx1 after immunoprecipitation of pTyr-Prdx1 from whole tissue lysate of EL-Kras and KC mouse pancreas. **(B)** Levels of Prdx1 bound to pERK after immunoprecipitation of pERK from whole tissue lysate of EL-Kras and KC mouse pancreas. **(C)** Prdx1 expression and oligomerization in whole tissue lysate from the same EL-Kras and KC mice after post-lysis treatment with DTT. (EL-Kras, n=5; KC, n=4.)

To further investigate the mechanism of Prdx1 and its post-translational modifications on pERK in EL-Kras and KC mice, we evaluated levels of Prdx1/pERK and pTyr-Prdx1/pERK interactions in snap frozen pancreas lysate from 7-8 month old EL-Kras and 6 month old KC mice (Figure 5). We also used DTT in non-reducing westerns with the same lysate to determine if Prdx1 oligomers are differentially oxidized in EL-Kras and KC pancreas. We found no significant differences in levels of Prdx1 and pERK interactions in EL-Kras mice and KC mice (Figure 5B), but there were significantly higher levels of pTyr-Prdx1/pERK interactions in EL-Kras mice as compared to KC mice (Figure 5A) (See Supplementary Figure 13). In addition, DTT down-shifted Prdx1 oligomers in non-reducing westerns to p25 Prdx1 (Figure 5C), indicating that higher molecular weight Prdx1 oligomers are indeed oxidized. Interestingly, as seen in Figure 4, p100 Prdx1 oligomers were only present in KC mice but not EL-Kras mice, indicating differential levels of Prdx1 post-translational modification and oligomerization in mice with different levels of Txn expression. Our data suggests that changes in Prdx1 post-translational modification could be associated with differences in its ability to interact with and regulate pERK signaling.

## DISCUSSION

Redox biology plays a significant role in tumorigenesis and improved understanding of its contribution to pancreatic carcinogenesis could improve therapy. Some antioxidant proteins, such as Prdx1, have the potential to be used as diagnostic and/or prognostic indicators. Increased Prdx1 expression in pancreatic cancer patient serum and tissue correlates with decreased overall and relapse-free survival and elevated VEGF expression [2, 3]. Understanding the mechanisms that regulate Prdx1 nuclear localization and/or secretion throughout pancreatic tumorigenesis will be essential to determining its role in pancreatic cancer aggressiveness and patient survival. Indeed, because higher nuclear Prdx1 expression was significantly associated with better survival in our study (Figure 1), cancers that maintain elevated nuclear Prdx1 levels may have more reduced Prdx1 available in the nucleus to suppress transcription factor DNA binding and activity [15, 16, 18]. Moreover, the association of lower nuclear Prdx1 levels with worse survival and potentially more lymph node involvement suggests that there is less reduced nuclear Prdx1 available to suppress tumorigenesis in patients with low nuclear Prdx1 in tumors. Additionally, our data suggests that during the earlier stages of neoplasia, the way Prdx1 functions and responds to oxidative stimuli is altered because it is post-translationally modified and the expression of Txn and Srxn is suppressed.

In addition to less Txn and Srxn expression, the more aggressive cells have higher levels of oligomerized Prdx1. We hypothesize that in the more aggressive human tumors with less nuclear Prdx1, Prdx1 has been hyperoxidized and secreted from cells throughout tumorigenesis. This hypothesis coincides with literature that shows that high serum levels of Prdx1 are associated with a worse prognosis and elevated VEGF levels in pancreatic cancer patients [2, 3]. Furthermore, Prdx1 can be secreted in response to hyperoxidation [20]. Therefore, understanding the role of Prdx1 in neoplasia and cancer may require determination of the mechanisms that regulate Prdx1 nuclear localization and secretion.

To determine if Prdx1 mRNA levels correlate with elevated Prdx1 expression in patient tumor samples, we used the Oncomine mRNA database of human normal and tumor samples. Differences in the mRNA expression of Nrf1 and Nrf2 were also evaluated to determine if changes in Prdx1 mRNA expression are associated with changes in the expression of these two major redox associated transcription factors that can induce the expression of Prdx1 and the Txn system by binding to the antioxidant response element (ARE) in their promoters [5, 29–33]. This is relevant to pancreatic cancer because mutant Kras can induce Nrf2 expression in pancreatic neoplasia [1]. Interestingly, we found that Prdx1 mRNA was significantly elevated in human pancreatic cancer tissue, which correlated with increased Nrf1 mRNA expression but not with Nrf2 mRNA expression. This observation warrants further investigation because both Nrf1 and Nrf2 regulate AREs and an increase in Nrf1 mRNA expression suggests that there is more Nrf1 available to regulate ARE transcription in pancreatic tumors. This could have interesting consequences on regulation of ARE mediated transcription in pancreatic cancer because Nrf1 can both support Nrf2 function and suppress ARE mediated transcription depending on the Nrf1 isoform being expressed [30, 33–35]. In addition to Prdx1 expression, this might affect Txn and Srxn expression. While Nrf2 expression may be elevated in pancreatic cancer because of differences in its post-translational regulation and degradation in cancer, changes in Nrf1 mRNA levels could indicate differences in its overall expression or the transcription of an Nrf1 splice variant [36]. This could also result in differential regulation of the expression of Nrf1 isoforms that alternatively regulate transcription [5, 34]. Further investigation will be required to determine the significance of Nrf1 signaling in pancreatic cancer.

**Figure 6:**
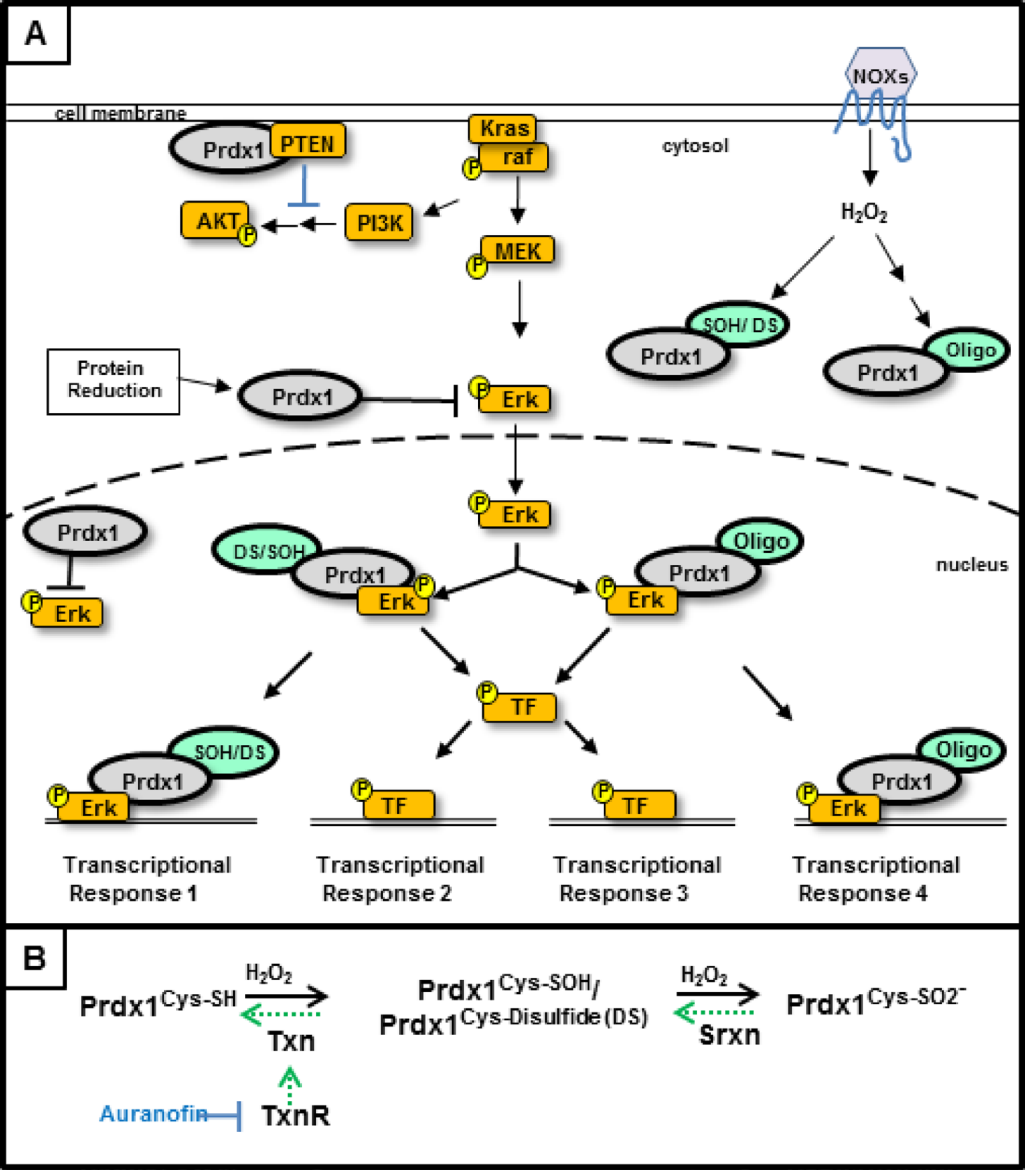
Regulation of Kras signaling by Peroxiredoxin-1- In our model of Kras associated disruption of the Txn system, Kras activated signaling results in decreased Txn and Srxn expression We propose that **(A)** decreased Txn and Srxn expression result in elevated levels of oxidized Prdx1 in the cell and decreased inhibition of ERK signaling by reduced Prdx1^Cys-SH^. Upon overoxidation and/or oligomerization, Prdx1's function changes and becomes tumorigenic by enhancing pERK related signaling. Prdx1^Oligo^ in this model represents higher molecular weight Prdx1 bands (p100, p250+). The effect of Prdx1 on AKT signaling may also decrease as a result of Prdx1's diminished redox function and its inability to reduce the active site of PTEN. **(B)** The redox cycle of Prdx1 and the Txn system. Reduction of Prdx1^Cys-SOH^ and Prdx1 disulfide (Prdx1^DS^) requires Txn to return Prdx1 to its reduced, Prdx1^Cys-SH^ state. Upon further oxidation to Prdx1^Cys-SO2-^ or when Txn expression is inhibited (i.e. Kras suppressed Txn expression or auranofin treatment), Srxn is required to reduce Prdx1. Auranofin inhibits the function of TxnR, which reduces oxidized Txn.

Maintaining the redox function of Prdx1 is critical for its tumor suppressive functions. Determining what alters the redox state and localization of Prdx1 during pancreatic tumorigenesis will be essential to understanding the effect of Prdx1 on patient survival. Oxidation of the redox active cysteines of Prdx1 (cysteine sulfenic acids (CSA) and Prdx1-disulfide) is reversed by Txn [14, 17, 23–26]. If Prdx1 is further oxidized (i.e. sulfinic acid), its redox function is inhibited and Srxn is required to recover Prdx1's antioxidant activity (Figure 6). Kras associated changes in the expression or function of Txn and Srxn throughout pancreatic tumorigenesis will therefore have a significant effect on Prdx1 signaling in pancreatic cancer. Our data in Figure 2 demonstrate that suppression of Txn is differentially regulated in lesions of EL-Kras and KC mice and that Srxn expression is significantly lower in lesions of both models. In the absence of sufficient Txn and Srxn to reduce Prdx1, Prdx1 in neoplastic lesions could be hyperoxidized, oligomerized, and/or functioning as a chaperone [9, 37]. Its hyperoxidation could also contribute to elevated Prdx1 secretion and the perpetuation of inflammation by secreted Prdx1 as the tumor progresses [20]. We hypothesize that accumulation of these post-translational modifications and non-redox associated functions as lesions progress leads to the eventual secretion of Prdx1, which is associated with a worse prognosis in pancreatic cancer [2, 3]. Studies on Txn expression in pancreatic cancer patients also support this hypothesis. A randomized phase II study of the Txn inhibitor PX-12 in pancreatic cancer patients was terminated early due to unexpectedly low baseline Txn levels and insufficient antitumor activity [38]. Another study suggested that Txn expression is elevated in pancreatic cancer patient serum, but we hypothesize that this is due to elevated Txn secretion from exosomes or secretory vesicles [39]. Therefore, changes in the tumorigenic properties of Prdx1 during pancreatic neoplasia may be associated with mutant Kras induced changes in Txn and Srxn expression.

Identifying the mechanisms that control the subcellular localization of Prdx1, Txn, and Srxn will be important in understanding the role of the Txn system in pancreatic tumorigenesis. The functions of these proteins can vary significantly in different compartments. Due to the presence of some nuclear Prdx1 in normal acinar cells (Figures 1-2), we hypothesize that the nuclear Prdx1 present in neoplastic lesions exists in both an oxidized/oligomerized and a reduced state [9, 15]. Prdx1 overoxidation can activate its nuclear localization and inhibit its redox function [9, 37, 40]. Prdx1's redox function is also inhibited by phosphorylation by Src, Mst1, Cdc2, and other kinases [40–43], resulting in altered Prdx1 peroxidase activity, oligomerization, and nuclear chaperoning. Src is associated with Kras signaling in pancreatic neoplasia and with tyrosine phosphorylation of Prdx1 at Tyr194, which inhibits Prdx1 redox function [43, 44]. Phosphorylation of Prdx1 at Thr90 also activates its nuclear chaperone function and suppresses its redox activity. These Prdx1 post-translational modifications will have significant effects on its chaperone and cofactor functions (e.g. pERK, NF-kB) because Prdx1 will not be able to suppress ERK and NF-kB transcriptional activity if it is not in a reduced and redox active state [15, 16]. Deciphering the mechanisms that designate the conditions in which Prdx1 functions as a peroxidase, a chaperone, or an inflammatory factor will be essential to understanding its role in pancreatic cancer. Dysregulated Txn system expression and post-translational modification during pancreatic neoplasia could therefore have significant implications on transcription factor and Kras effector activity in pancreatic cancer.

To determine how oxidation of the Txn system regulates the phosphorylation of Kras effectors in pancreatic neoplasia, we treated EL-Kras and KC primary pancreatic cells with auranofin. We found that the ability of auranofin to affect ERK and AKT phosphorylation in primary pancreatic acinar cells (Figure 4) correlated with its ability to affect Prdx1 levels. Auranofin induced significant increases in p40 Prdx1 levels in EL-Kras cells, but p40 Prdx1 was unaffected by auranofin in KC cells. Moreover, similar to the p40 Prdx1 response, auranofin induced an increase in ERK phosphorylation in EL-Kras cells, but not in KC cells. By contrast, auranofin increased AKT phosphorylation in EL-Kras mice, but decreased AKT phosphorylation in KC mice. Interestingly, the p100 Prdx1 band was only present in KC mice. Although the p40 homodimer has previously been demonstrated in the literature, the makeup of the p100 Prdx1 oligomer seen in our study is unknown. We hypothesize that this p100 Prdx1 is either a partial decamer, with disulfide bonds at Cys83, or an oligomer of Prdx1 homodimers and another redox regulated protein [20]. The presence of this p100 Prdx1 in KC mice and the unresponsiveness of p40 Prdx1 to auranofin suggests that Prdx1 in cells from KC mice has an altered ability to oligomerize (which is also associated with an inability to respond to redox stimuli), despite there being some Txn expression in normal acinar cells in primary culture. Our *in vitro* data in Figure 3 suggests that the metastatic AsPC1 cells also have more Prdx1 oligomerization than the primary tumor Panc1 cells and the more normal and Kras mutant ductal cell lines, HPDE and HPDE-Kras. Due to the aggressive nature of the KC cells and the AsPC1 cells our data has significant implications on the role of Prdx1 post-translational modifications and oligomerization on the aggressiveness of this disease. Future studies investigating how changes in Prdx1 oligomerization, localization, and secretion throughout tumorigenesis affect the activity of Kras effectors will be important in understanding the effect of Prdx1 on patient survival.

Previously, it was demonstrated that Prdx1 suppresses the activity of redox sensitive ERK in a cyclin D1- dependent manner [18]. The effect of Prdx1 oxidation, oligomerization, or phosphorylation on the regulation of ERK, however, has not been investigated. We therefore investigated pERK/pTyr-Prdx1 and pERK/Prdx1 interactions in tissue from EL-Kras and KC mice to further evaluate the effects of modified Prdx1 post-translational regulation and function on ERK signaling in these mice (Figure 5). While there were no significant differences in pERK and total Prdx1 interactions in EL-Kras mice vs. KC mice (Figure 5B), there was significantly more pERK bound to pTyr-Prdx1 in EL-Kras mice than in KC mice (Figure 5A). In addition, our preliminary data in these mice suggest that p21 is also bound in a complex with both pERK and pTyr-Prdx1 (data not shown). Although the mechanism of pTyr-Prdx1 in pancreatic neoplasia is unknown, this suggests that Prdx1 might also be involved in regulation of the cell cycle in pancreatic cells. Interestingly, previous studies on pTyr-Prdx1 suggest that (although Prdx1 redox function is inhibited by tyrosine phosphorylation) its redox function can be partially restored by Txn mediated reduction of pTyr-Prdx1 [43]. Our data suggests that Txn expression is significantly lower in lesions of KC mice than in lesions of EL-Kras mice. The ability of Prdx1 to affect pERK signaling could therefore be differentially regulated by Prdx1 Tyr phosphorylation, Prdx1 oligomerization, and the presence of Txn to restore Prdx1's function. To further support this hypothesis, the p100 Prdx1 oligomer found in KC mice is not present in EL-Kras mice (Figures 4 and 5) and DTT was unable to completely reduce Prdx1 oligomers in whole tissue lysate from KC mice (Figure 5C). This implies that pTyr-Prdx1 in KC mice is more post-translationally modified than pTyr-Prdx1 in EL-Kras mice and less capable of performing its normal tumor suppressive antioxidant functions. Future investigation of the association between these modifications and the modified localization and/or secretion of Prdx1 could also have significant implications on the prognosis of pancreatic cancer.

This novel study provides a foundation for future studies on the redox biology of pancreatic tumorigenesis. It will be important to better understand how the Txn system regulates pancreatic tumorigenesis and what transcription factors (Nrf1 and Nrf2) and signaling proteins (ERK) control changes in Prdx1 function. Determining the timing of Prdx1 and/or Txn secretion during neoplasia could be a valuable diagnostic tool when used in combination with pancreatic cancer markers such as CA19-9. Understanding how Kras is associated with Prdx1 post-translational modifications that result in Prdx1 secretion and how Kras is associated with suppression of Txn and Srxn expression will provide insight into the role of the Txn system in this disease. Investigating the role of Prdx1 post-translational modifications in nuclear chaperoning and transcription will also be essential to understanding how Prdx1 signaling is involved in pancreatic tumorigenesis. We therefore propose that therapies designed to target Txn system post-translational modifications will improve pancreatic cancer therapy.

## MATERIALS AND METHODS

### *In vitro* cell culture and reagents

Normal Human Pancreatic Ductal Epithelial (HPDE) cells and mutant Kras HPDE cells (HPDE-Kras) were obtained from the Tsao lab [45, 46]. Panc1 (primary tumor cells) and AsPC-1 (metastatic cells) were obtained from ATCC. HPDE and HPDE-Kras cell lines were cultured and treated in low serum keratinocyte serum free media (supplied supplements- 1ml bovine pituitary extract, 1ul EGF) (Gibco, Waltham, MA, Cat#10724-011). Panc1 and ASPC-1 cells were cultured in full DMEM media (10% FBS) and treated in serum free DMEM. All *in vitro* and primary culture treatments were 24 hrs. Auranofin was from Sigma (St. Louis, MO) (1uM Auranofin).

Antibodies- Western and IP: Peroxiredoxin-1 (Abcam, Cambridge, MA, Cat# 59538), Phospho-Prdx1 (Tyr194) (Cell Signaling (CST), Danvers, MA, Cat#14041), Thioredoxin-1 (CST Cat#2429), Sulfiredoxin (Proteintech group (PTG), Rosemont, IL, Cat#14273-1-AP), anti-dimedone (CSA) (Millipore, Billerica, MA, Cat# 07-2139); pERK, ERK, pAKT, AKT (CST), GAPDH (Santa Cruz Biotechnology, Dallas, TX), Conformation specific secondary anti-rabbit IgG (HRP conjugated) (CST Cat#5127). IF and IHC: Peroxiredoxin-1 (Abcam Cat#41906), Anti-Thioredoxin / TRX antibody (Abcam Cat#86255), Sulfiredoxin (PTG Cat# 14273-1-AP), CK19 (Cytokeratin 19) -TROMA III Ab (DSHB, University of Iowa, IA).

### Primary cell culture

We used a modified pancreatic acinar isolation protocol [47–49]. We removed the pancreas from five month old EL-Kras [50] and KC mice, rinsed it in 1x HBSS with 10mM HEPES (HBSS), then minced the pancreas into 10ml of 250 ug/ml Collagenase Type I (Gibco) and 250 ug/ml Dispase II (Sigma D4693) in HBSS. After transfer into a T-25 flask, the solution was pipetted up and down 10 times every 5 minutes with a 10-25ml pipette and returned to the 37°C incubator. This continued until the solution was opaque and minimal large pieces remained (∼30 mins). Once cells were in solution, enzyme was inactivated with 10 ml cold 30% FBS in HBSS. The solution was passed through a 100um filter into a 50 ml tube and remaining tissue in the filter was gently pressed through and rinsed with 10mls cold 5% FBS in HBSS. After an additional wash and pressing, the cells were spun down at 1000xg for 10 mins and supernatant was removed. The pellet was washed again after centrifugation in cold 5% FBS in HBSS before resuspension and plating in full Waymouth's media (see below). The next day, floating, non-attached cells were centrifuged and replated in low serum Waymouth's media (see below) on top of a thin layer of solidified Collagen Type1 (Gibco 17100-017)/ 1X MEM in a 6 well plate. After overnight serum starvation in low serum media, cells were treated with Auranofin and/or 100 ng/ml TGF-α (Affymatrix eBioscience, San Diego, CA, 34-8698-82) for 24 hrs. Cells were isolated with 1 mg/ml collagenase type 1 in HBSS then rinsed twice in HBSS at low speed before lysis in redox lysis buffer (see Immunoblotting) for westerns.

### Full Waymouth's media

Waymouth's media (Gibco), 2.5% FBS, 250 ug/ml soybean trypsin inhibitor (Life Technologies), 25 ng/ml EGF (Gibco PHG0311L), 1 mg/ml Dexamethasone (Sigma), 10mM HEPES, 0.26% Sodium Bicarbonate, and Gentamicin. Low serum Waymouth's media- Full Waymouth's media with 0.25% FBS and no dexamethasone.

### Transgenic mice and patient samples

We used 6 month old p48-Cre/LSL-Kras mice (KC) and 12 (IF) month or 8 month (pancreatic lysate) old EL-Kras mice [50]. Animals were bred at the University of Illinois-Chicago according to IACUC approved procedures. Patient samples were obtained from Dr. David Bentrem in the Department of Surgery at Northwestern University. All samples are IRB exempt. 60 patient sample pairs of normal and tumor tissue were used for this study.

### Immunohistochemistry and immunofluorescence

After deparaffinization, antigen retrieval was performed using DAKO antigen retrieval solution (DAKO S1699) in a pressure cooker (10 mins, 120°C; 10 mins, 90°C). After cooling, slides were washed in TBST and permeated (0.1% Triton X-100 in TBS; 10 mins, RT), then washed in PBS. Peroxidases were quenched in 3% hydrogen peroxide in methanol for 30 mins and washed in TBST. After blocking in Background Buster (Innovex Biosciences) for 30 mins, slides were incubated in 1:200 primary antibody in 0.5% BSA overnight at 4°C. Slides were then washed in PBS, DAKO Wash Buffer (DAKO S3006), and PBS. IHC: DAKO secondary antibody (anti-rabbit IgG) HRP-linked (DAKO P0448) was added for 30 min at RT or IF: Donkey anti-rabbit Alexa Fluor 594 secondary antibody (Abcam ab150064) in 0.5% BSA for 1 hr at RT. Slides were then washed with TBST before DAB development and counterstaining (IHC) or mounting with ProLong Gold Antifade Mountant with DAPI (ThermoFisher Scientific) (IF). Representative IF pictures shown were minimally manipulated in an approved manner to minimize low level, non-specific background levels in merged pictures.

### Survival human tumor array and statistical analysis

The tissue microarray (TMA) of AJCC stage I/II resected pancreatic adenocarcinoma has been previously described [51]. (See Supplementary Figures 1-2) Immunohistochemistry was visualized using the Dako Envision+ System- HRP (K4002), after de-paraffinization, heat-induced antigen-retrieval with vegetable steamer for 20 minutes in citrate buffer (pH 6.0) and one hour incubation at room temperature with anti-PRDX1 primary antibody (Abcam 41906) at 1:100 dilution. Three separate 1.0 mm cores for each tumor in the TMA were scored for nuclear and cytoplasmic expression by a blinded observer using semi-quantitative histoscores (range 0-300), representing the product of staining intensity (0-3) and percentage of tumor cells staining at that intensity (0-100). Separate scores were generated for nuclear expression and cytoplasmic expression in each core. For final dichotomization, each tumor was assigned into either a low or high level staining group based on its median histoscore. Survival estimates were generated using the Kaplan-Meier method and compared using log-rank tests. Multivariate Cox proportional hazards models were used to test statistical independence and significance of multiple predictors with backward selection performed using the Akaike Information Criterion. Overall survival time was measured from the date of surgery to the date of death due to any cause or right-censored based last date of clinical follow-up.

### Western blotting and immunoprecipitation

Whole tissue pieces or cells were scraped into degassed redox lysis buffer (100mM Tris pH 7.4, 1% Triton X-100) containing 10mM Dimedone (Sigma 38490) for sulfenic acid labeling. Following sonication and a 40 min incubation on ice, N-Ethyl maleimide (NEM) (Sigma E3876) was added (final concentration 100mM) for 15 mins before centrifugation. For non-reducing westerns of redox proteins and oxidative modifications (Prdx1, Txn, and Srxn, and CSA), non-denatured lysate with 5x loading dye containing 100 nM NEM (65.79 mM Tris HCl, 21% glycerol, 0.003% Bromophenol blue, 4.21% SDS) was loaded directly. All other proteins for westerns and immunoprecipitations were boiled in and loaded in 4x laemmli buffer with 10% β-mercaptoethanol. Immunoprecipitations were performed by incubating protein with antibody overnight at 4° in redox lysis buffer before a 2 hr incubation with Protein A/G Plus agarose beads (SCT) and boiling in 4x laemmeli/BME buffer.

### RT-PCR

mRNA isolation: Trizol reagent (Invitrogen). cDNA: High Capacity Reverse Transcription kit (Applied Biosystems). PCR: GoTaq Green Master Mix (Promega), RT-PCR primers (Integrated DNA Technologies (Coralville, IA)): Prdx1: 5′-GTCCCACGGAGATCATTGCTTTC -3′ and 5′-CCCCTGAAAGAGATACCTTCATC-3′; GAPDH: 5′-TCCCATCACCATCTTCCA-3′ and 5′-CATCACG CCACAGTTTCC-3′; Fold changes were calculated after normalization to GAPDH levels.

### Oncomine

We used the online database Oncomine to search previously published data sets of mRNA expression in human pancreatic cancer patients. Using the Logsdon study dataset [27], in which samples were microdissected for specificity, we compared mRNA levels of genes in normal tissue and in pancreatic cancer tissue (Normal, n=5; Tumors, n=10). In the original manuscript, the authors paired groups from normal, tumor, and pancreatitis samples using resulting simple contrast tests that are similar to standard two-sample T-tests where the variance is estimated from all three groups. Fold changes are expressed by replacing mean expression values greater than 100 as 100 to avoid spurious values.

### Statistical analysis

All RT-PCR, western, and immunoprecipitation experiments were done at least 3 times. All IHC and IF pictures are representative of at least 5 mice in each genotype. Pictures from the first human patient sample tumor array are representative of 60 case pairs of normal and tumor samples. All data are expressed as the mean ± SD. All statistical tests were 2-sided. P-values of less than 0.05 were considered statistically significant. For each measurement variable, the distribution of normality was conducted before statistical analysis of testing group differences. We chose either parametric or non-parametric version of statistical procedure as appropriate based on the data distribution.

Differences between two groups were analyzed using two-sample Student's t-test or Wilcoxon rank sum test; differences among more than two groups were analyzed using one-way ANOVA or Kruskal-Wallis test as appropriate. Multiple comparisons of mean post hoc test for one-way ANOVA were adjusted using Tukey's method [52], while multiple comparisons of mean ranks post hoc test with a Kruskal-Wallis analysis were adjusted using Nemenyi (Tukey-type) test (P. Nemenyi, Distribution-Free Multiple Comparisons, State University of New York, Downstate Medical Center, 1963 (cited in Wilcoxon and Wilcox, 1964)). For equal group sizes or Dunn's test [53]. For unequal group sizes or some ranks tied [54]. Statistical analyses were performed using SAS version 9.4 (SAS Institute, Inc., Cary, NC, USA).

## SUPPLEMENTARY MATERIALS FIGURES AND TABLES


