# Supplementary material for: Thioredoxin system-mediated regulation of mutant Kras associated pancreatic neoplasia and cancer
Source: Oncotarget. 2017 Oct 4;8(54):92667–81. doi: 10.18632/oncotarget.21539 (PMC5696213; doi:10.18632/oncotarget.21539)
Supplement: Supplementary file 1 [file oncotarget-08-92667-s001.pdf]

Thioredoxin system-mediated regulation of mutant Kras associated pancreatic neoplasia and cancer

SUPPLEMENTARY MATERIALS

A

Variable	Overall Survival		
	Adjusted HR* (95% CI)	p-value†	
High Prdx1 Nuclear Histoscore	0.49 (0.29 – 0.82)	0.006	
High histologic grade	2.14 (1.42 – 3.24)	0.0003	
Lymph node involvement (pN1)	1.75 (1.16 – 2.64)	0.008	
High pT stage (pT3)	1.69 (1.12 – 2.57)	0.013	

B

	Median survival (95% confidence interval) (months)
Prdx1 Nuclear Histoscore 0–199	20.4 (13.4 – 27.4)
Prdx1 Nuclear Histoscore 200–300	43.92 (31.6 – 56.2)

Log-Rank Test P=0.001

Supplementary Figure 1: (A) Multivariate proportional hazards analyses of overall survival in pancreatic cancer TMA *HR (hazard ratio): HR of 1 indicates no difference between the two groups of patients for the listed variable, while a HR > 1 indicates an increased risk of death/failure for the group listed. †p-value from Chi-square test using the Cox proportional hazards model. The following covariates were initially evaluated in a Cox proportional hazards model with backward selection: 1. Nodal involvement (No vs. Yes), 2. Tumor Differentiation (Well-Moderate vs. Poor), 3. T-stage (T1-T2 vs. T3), 4. Tumor size (<3 vs. ≥3 cm), 5. Age (<60 vs. ≥60 yrs), 6. Gender, 7. Surgical Margins (Negative vs. Positive), 8. Prdx1 Nuclear Histoscore (0-199 vs. ≥200). **(B)** Kaplan-Meier Curves (descriptors).

C

Clinicopathologic Category	Prdx1 Nuclear Histoscore		
	Low (0-199)	High (200-300)	P value[a]
Total patients (n)	106	33	N/A
Age (years)			
< 60	34	13	0.28
≥ 60	72	20	
Gender			
Male	53	19	0.29
Female	53	14	
Histologic grade[c]			
Low	61	19	0.58
High	45	14	
T-stage (categorized)			
T1+T2	61	20	0.46
T3	45	13	
N-stage (categorized)			
N0	46	20	0.06
N1	60	13	
AJCC Stage			
I	27	13	0.10
II	79	20	
Surgical Margins			
R0	93	30	0.44
R1	13	3	
Tumor size (categorized)			
< 3 cm	64	20	0.57
≥ 3 cm	42	13	

Supplementary Figure 2 (*Continued*): (C) Clinicopathologic characteristics and group membership in UCLA Stage I/II pancreatic cancer TMA.

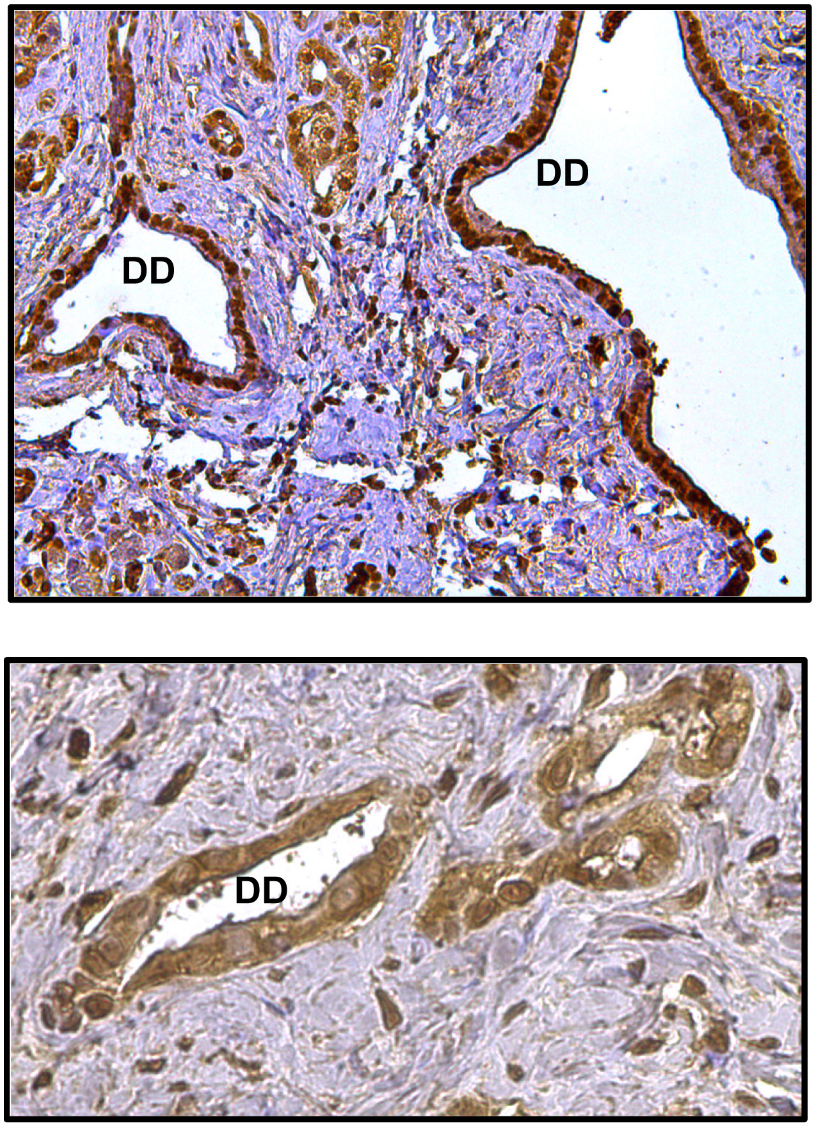

Supplementary Figure 3: Ductal Prdx1 staining (by IHC) in dysplastic ducts of human pancreatic cancer patients.

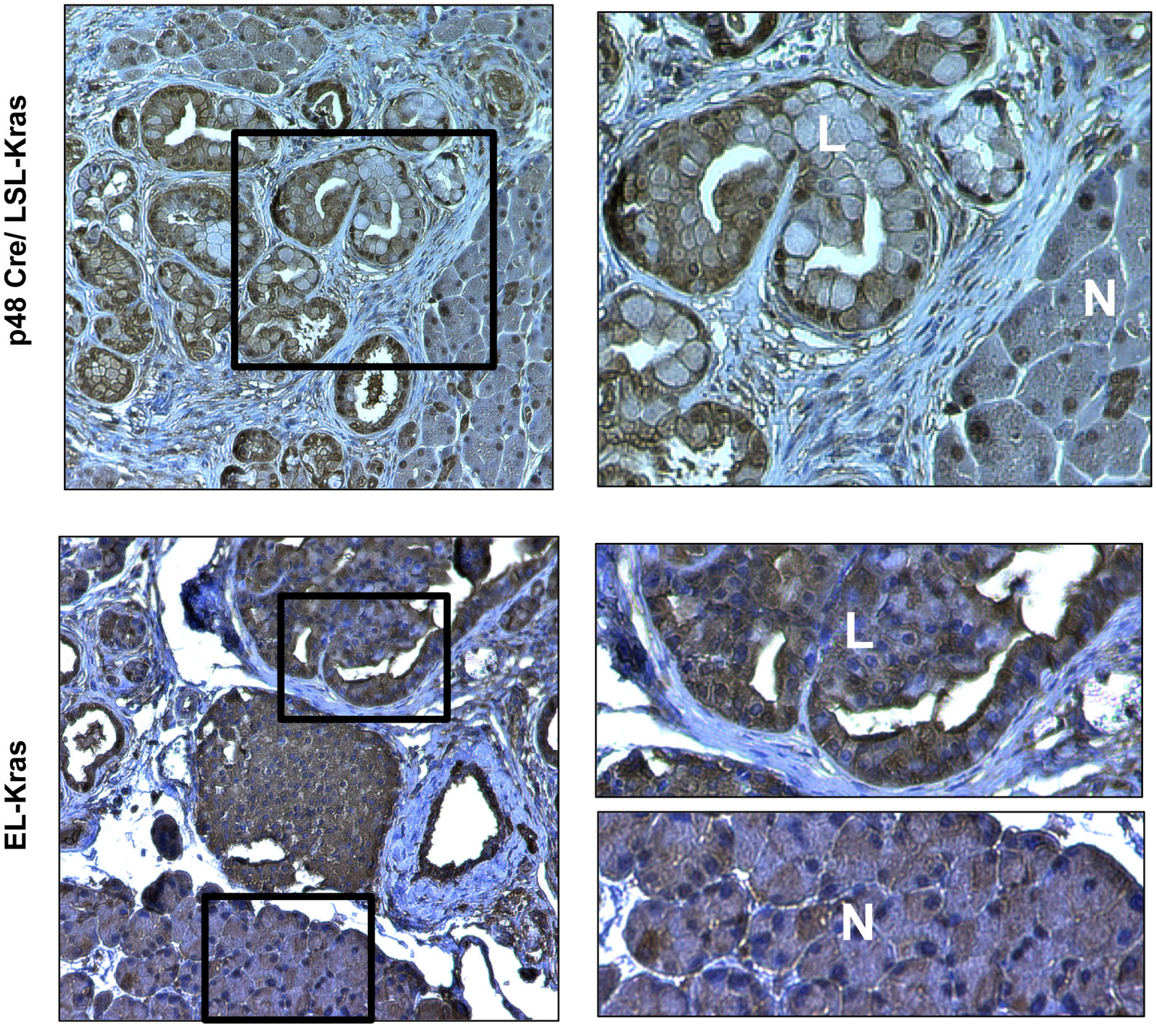

Supplementary Figure 4: (Left Column) 40x pictures of Prdx1 staining by IHC in EL-Kras and KC mice from Figure 2. (Right Column) Expanded pictures of Prdx1 staining in Normal (N) pancreatic tissue and Lesions (L) pictures taken in the left column.

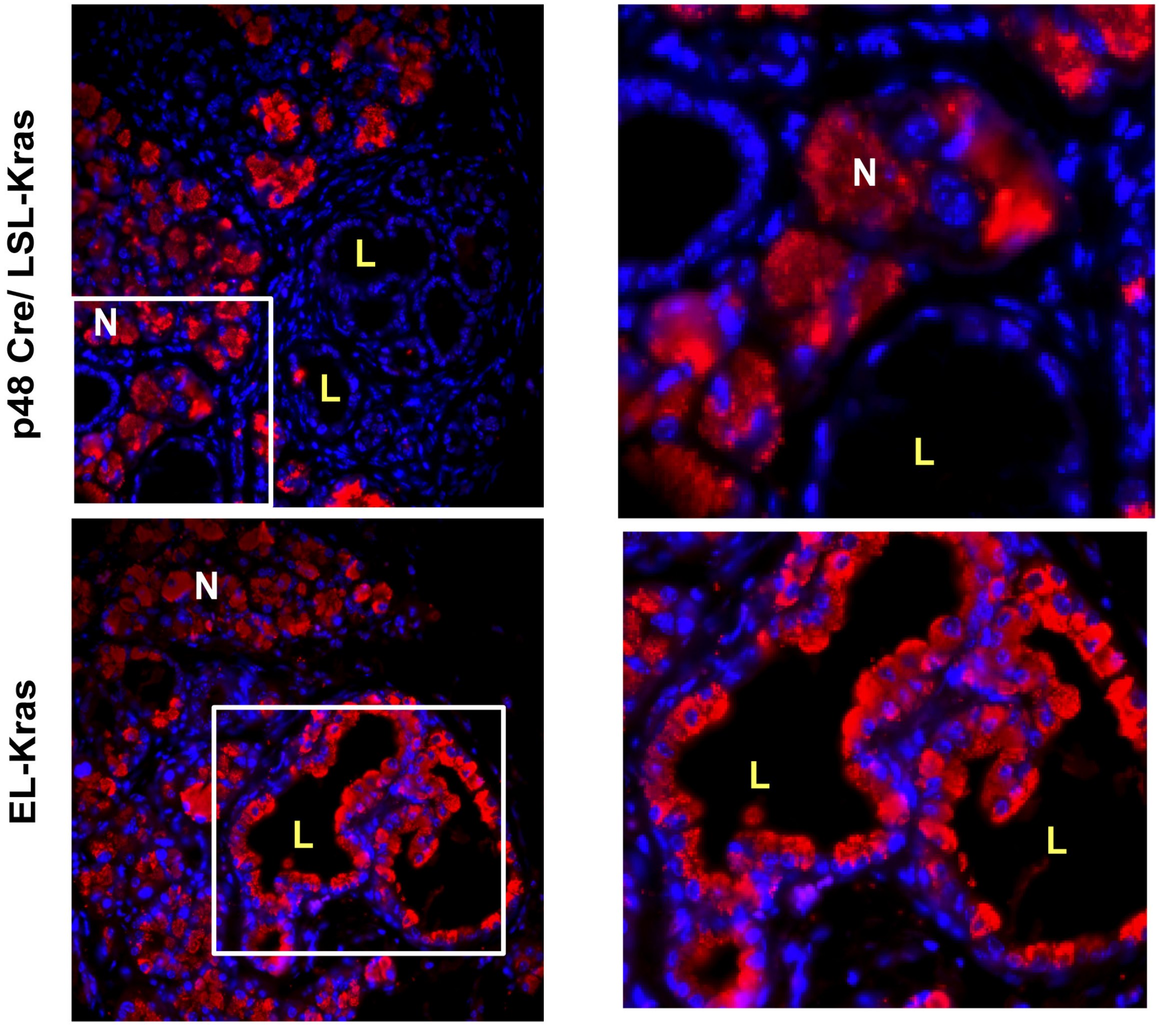

Supplementary Figure 5: (Left Column) 40x pictures of Txn staining by IF in EL-Kras and KC mice from Figure 2. (Right Column) Expanded pictures of Txn staining in Normal (N) pancreatic tissue and Lesions (L) pictures taken in the left column.

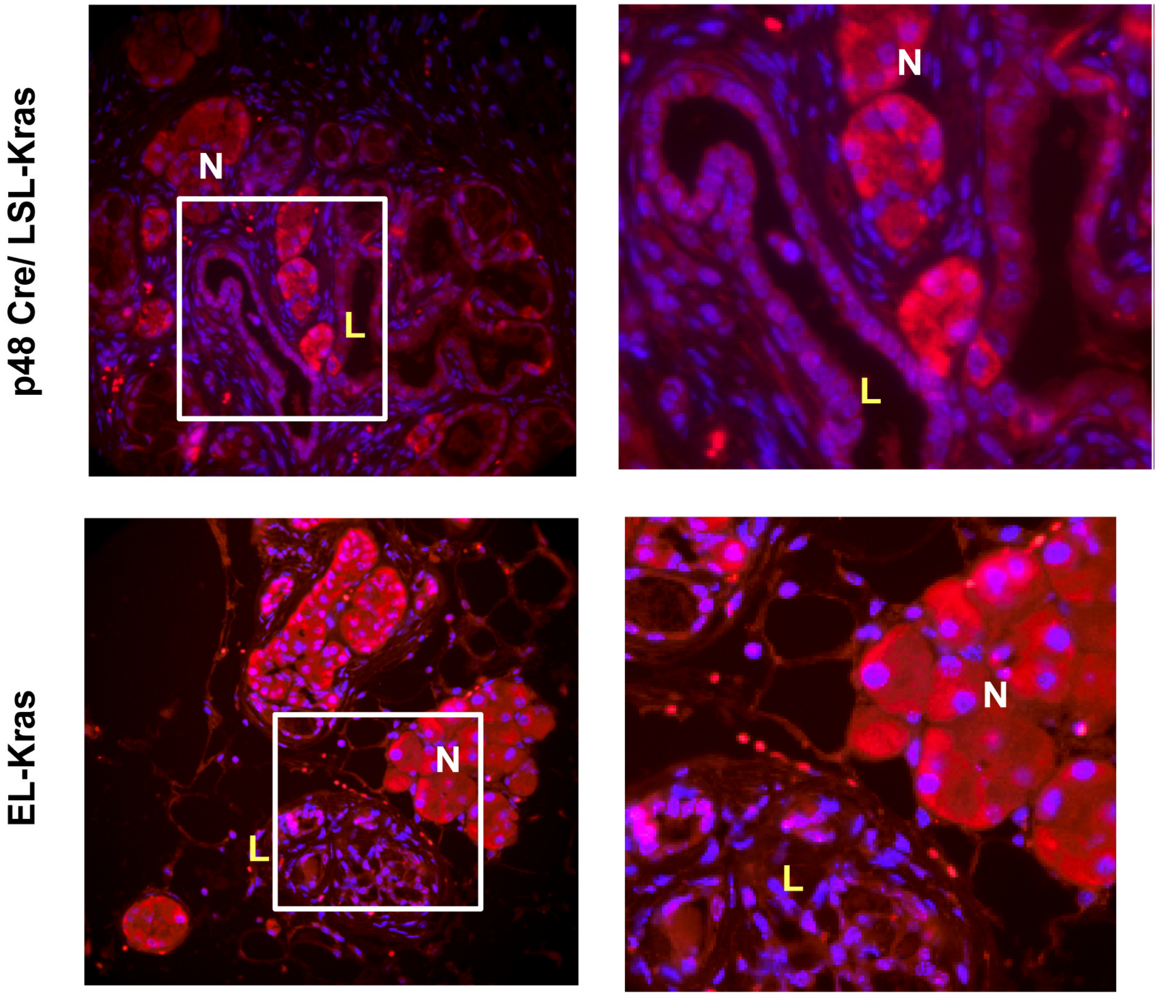

Supplementary Figure 6: (Left Column) 40x pictures of Srxn staining by IF in EL-Kras and KC mice from Figure 2. (Right Column) Expanded pictures of Srxn staining in Normal (N) pancreatic tissue and Lesions (L) pictures taken in the left column.

A. EL-Kras mouse : Txn (Red), CK19 (Green), DAPI (Blue)

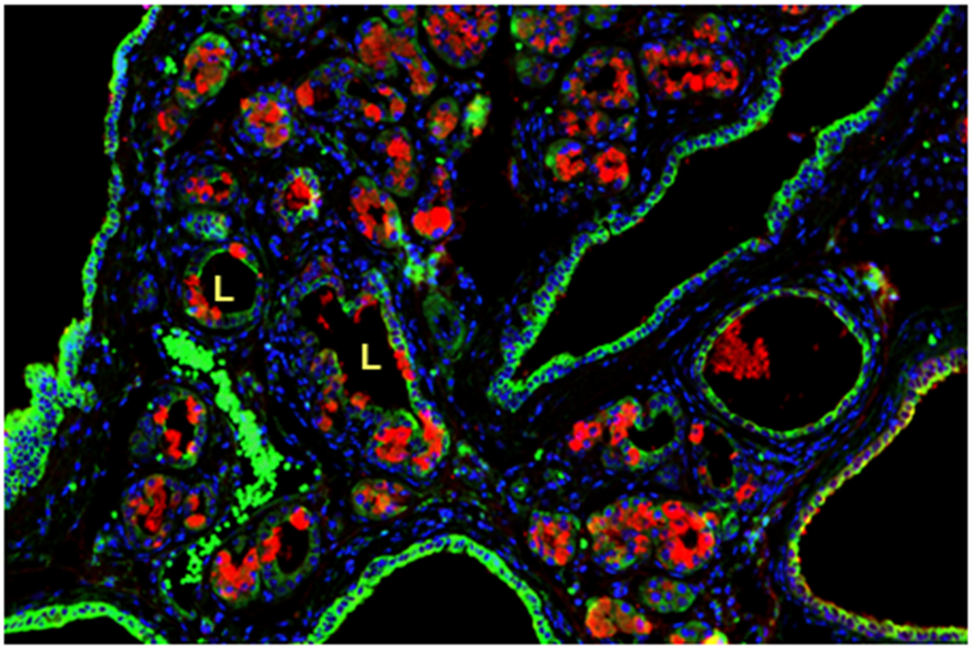

B. KC mouse: Txn (Red), CK19 (Green), DAPI (Blue)

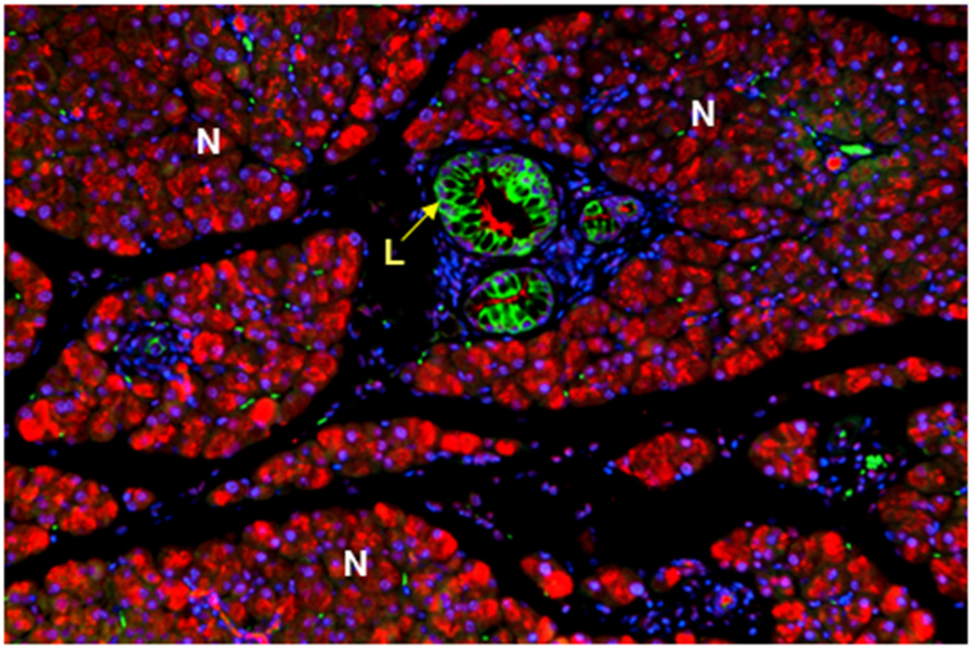

Supplementary Figure 7: Txn (red) and CK19 (green) co-staining by IF in pancreas of EL-Kras and KC mice.

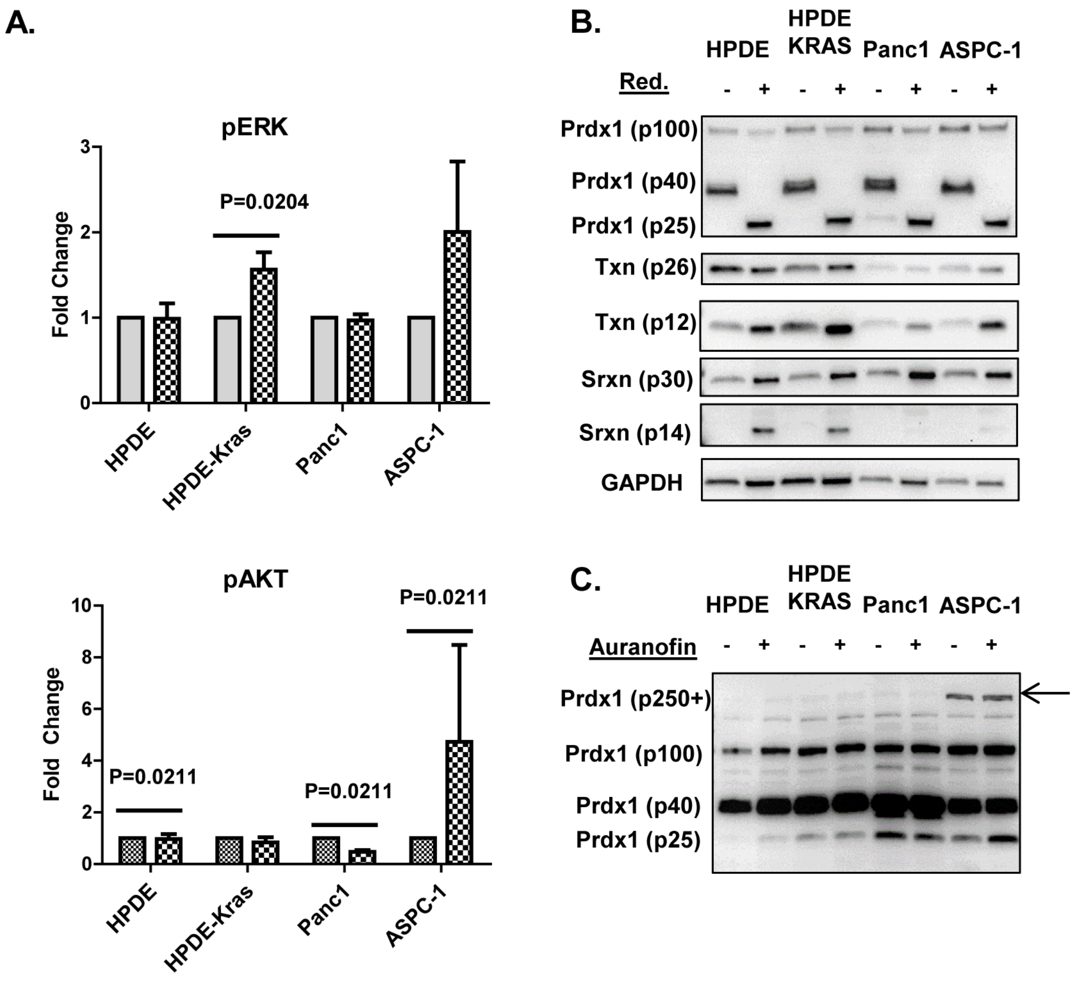

Supplementary Figure 8: (A) Statistical analysis for pERK and pAKT levels in auranofin treated pancreatic cells *in vitro* in Figure 3B. **(B)** Prdx1, Txn, and Srxn migration patterns in non-reducing and reducing westerns from untreated pancreatic cell lines *in vitro*. **(C)** Representative picture of the high MW (250+ kD) Prdx1 oligomer in AsPC1 cells *in vitro*. in auranofin treated cells.

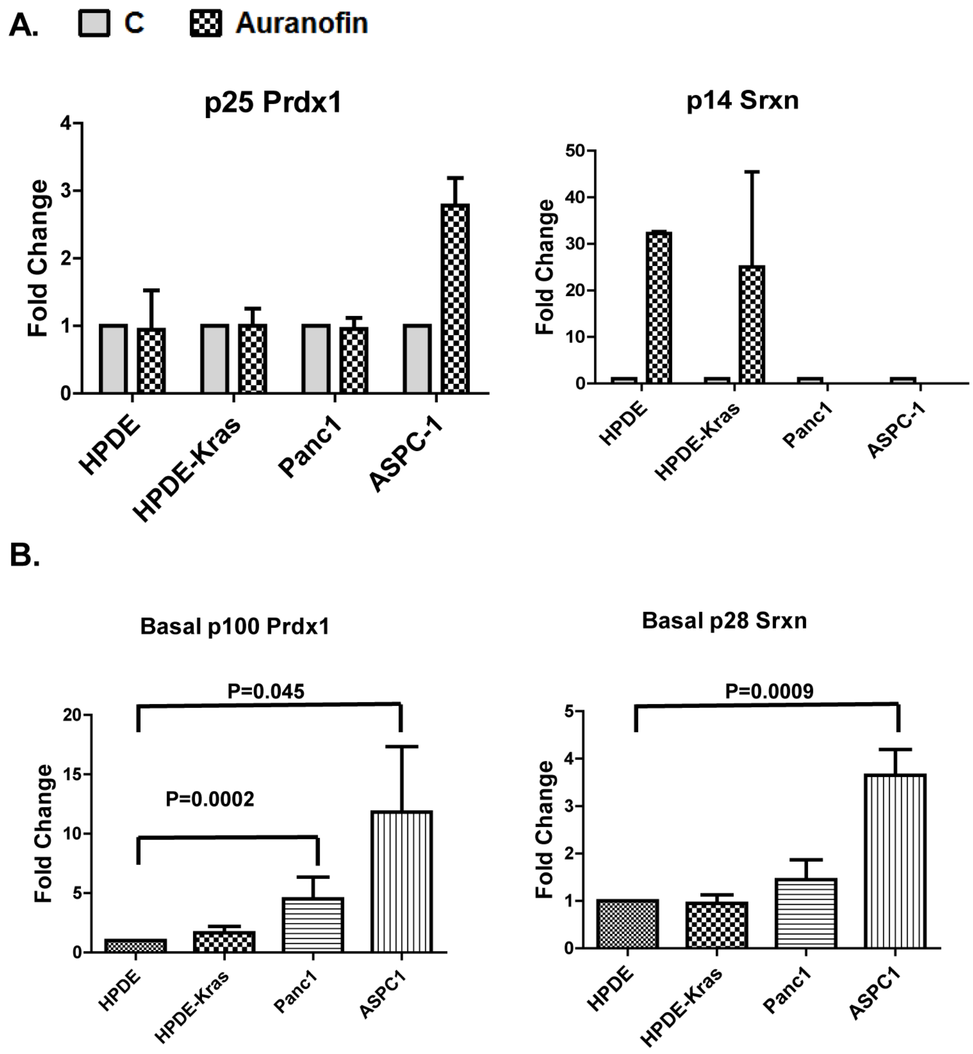

Supplementary Figure 9: Statistical analysis of Figure 3 Prdx1 data and Srxn data shown in Supplementary Figure 12.

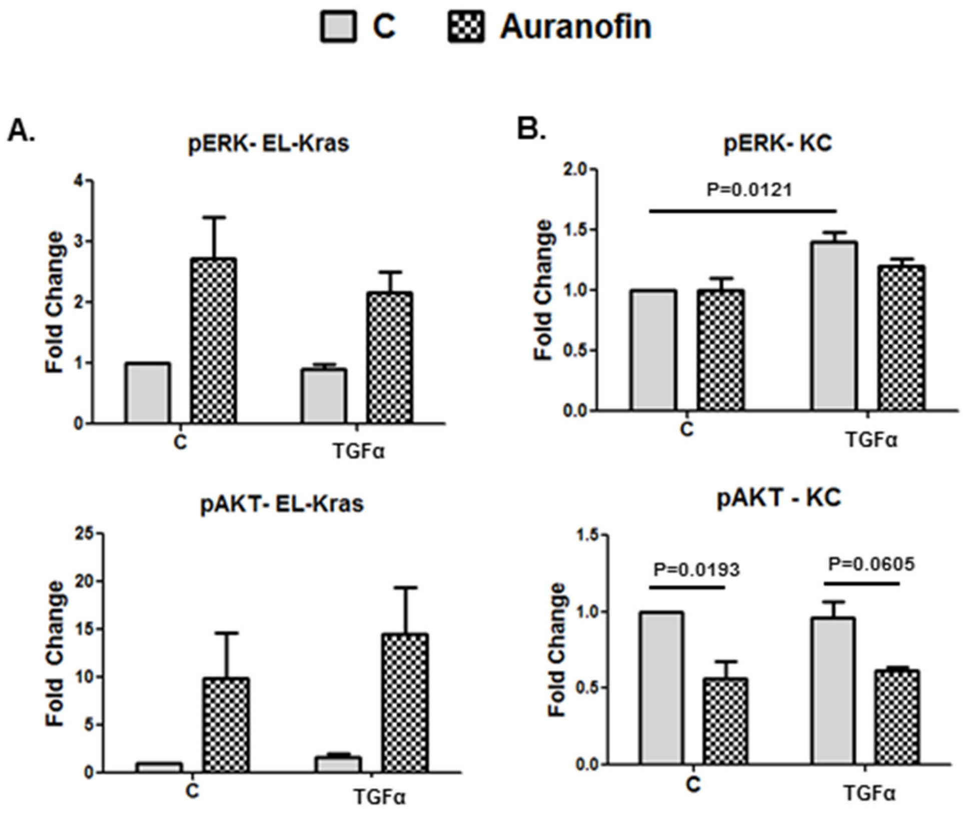

Supplementary Figure 10: Statistical analysis of pERK and pAKT protein expression levels of primary culture western data shown in Figure 4.

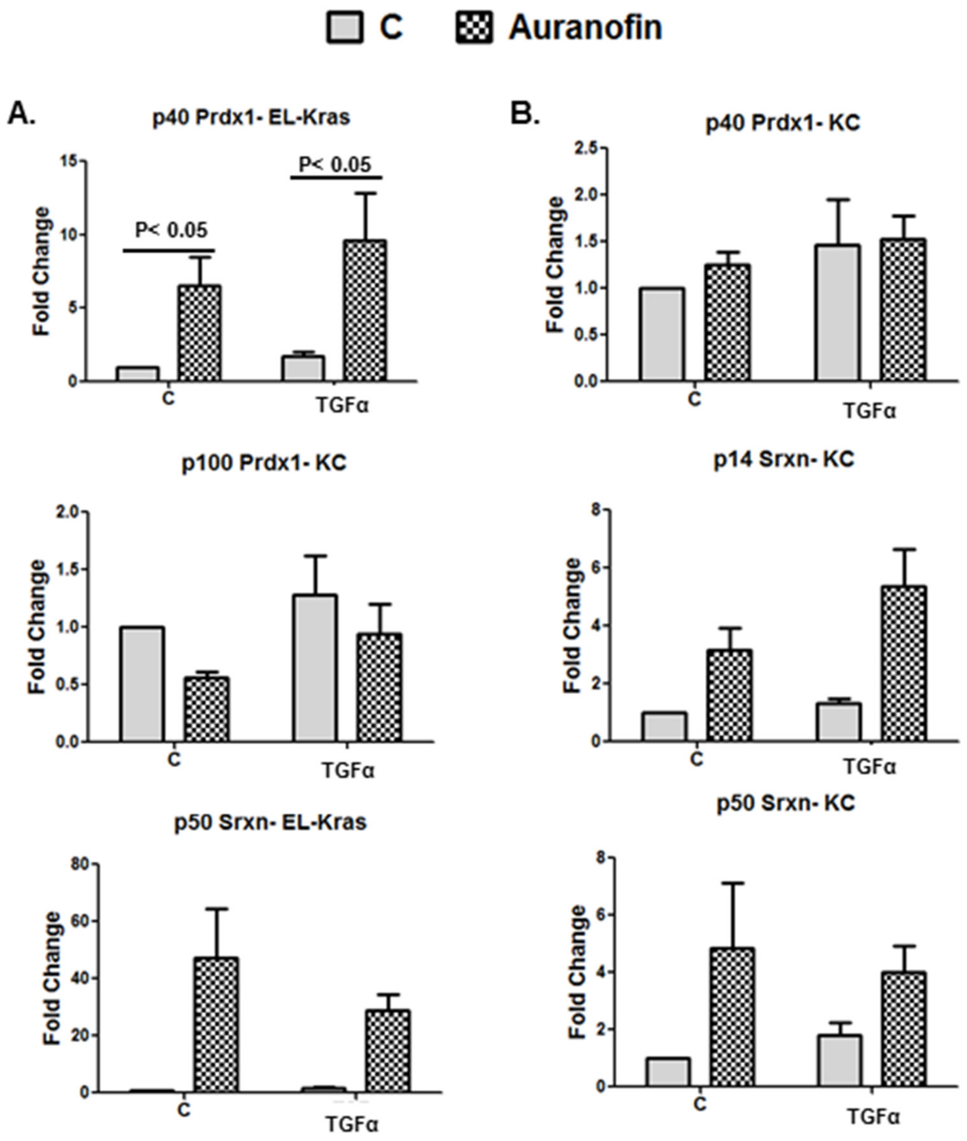

Supplementary Figure 11: Statistical analysis of Prdx1 and Srxn protein expression levels of primary culture western data shown in Figure 4 and Supplementary Figure 12.

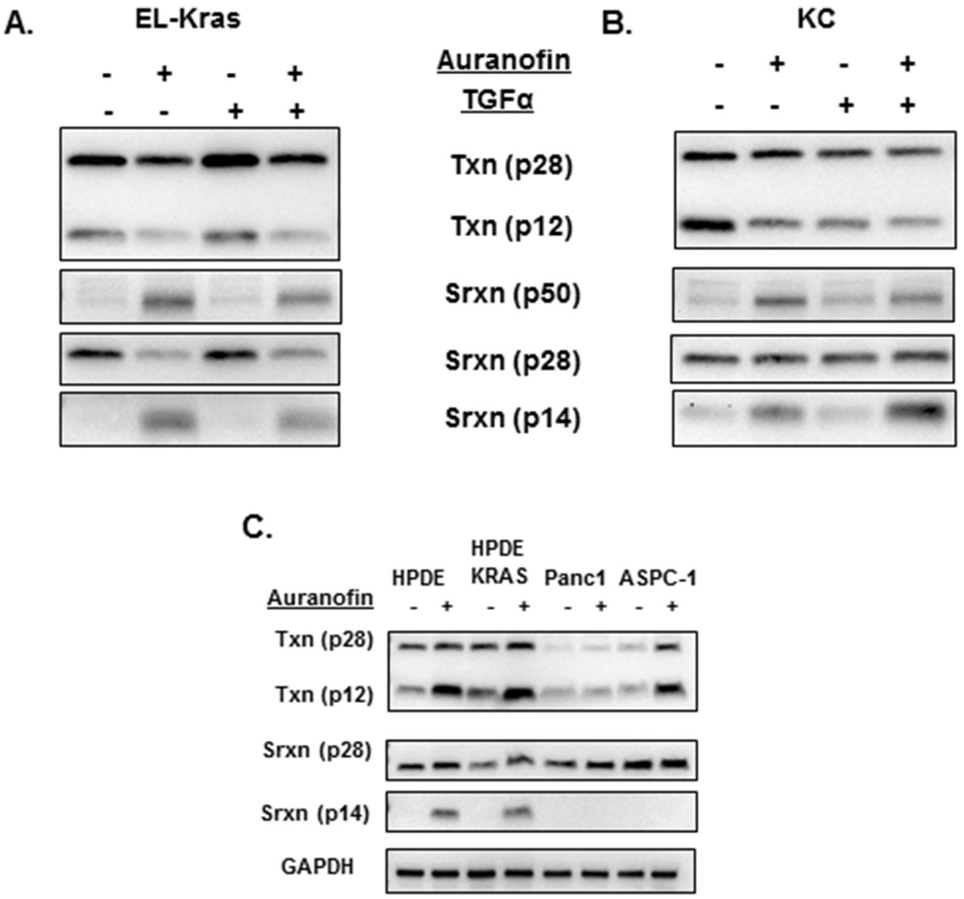

Supplementary Figure 12: (A-B) Txn and Srxn expression in pancreatic primary culture lysates from EL-Kras (A) and KC (B) mice. **(C)** Auranofin's effect on Txn and Srxn expression in pancreatic cell lines *in vitro*.

A. pTyr Prdx1 IP, pERK IB

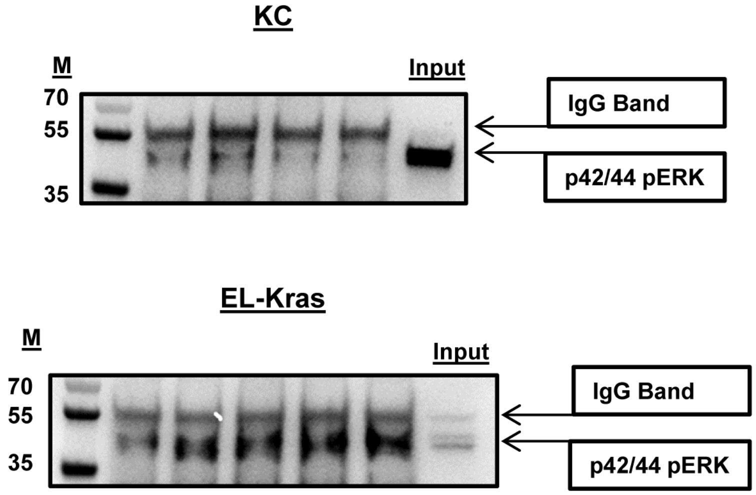

B. pERK IP, Prdx1 IB

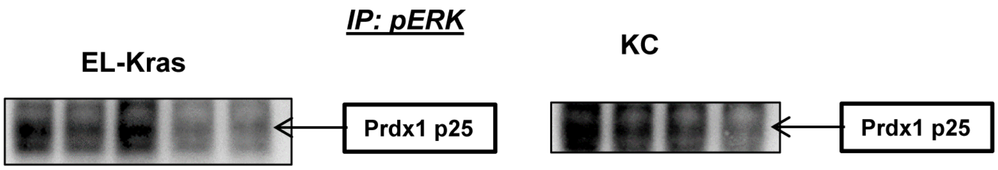

Supplementary Figure 13: (A) Expanded pictures of immunoprecipitations done in Figure 5A (A) and Figure 5B **(B)**.
